# Zika virus in southeastern Senegal: survival of the vectors and the virus during the dry season

**DOI:** 10.1186/s12879-020-05093-5

**Published:** 2020-05-24

**Authors:** Babacar Diouf, Alioune Gaye, Cheikh Tidiane Diagne, Mawlouth Diallo, Diawo Diallo

**Affiliations:** Pôle de Zoologie Médicale, Institut Pasteur de Dakar, 36 Avenue Pasteur, BP 220 Dakar, Senegal

**Keywords:** Zika virus, *Aedes*, Eggs, Vertical transmission, Local maintenance, Southeastern Senegal

## Abstract

**Background:**

Zika virus (ZIKV, genus *Flavivirus*, family *Flaviviridae*) is transmitted mainly by *Aedes* mosquitoes. This virus has become an emerging concern of global public health with recent epidemics associated to neurological complications in the pacific and America. ZIKV is the most frequently amplified arbovirus in southeastern Senegal. However, this virus and its adult vectors are undetectable during the dry season. The aim of this study was to investigate how ZIKV and its vectors are maintained locally during the dry season.

**Methods:**

Soil, sand, and detritus contained in 1339 potential breeding sites (tree holes, rock holes, fruit husks, discarded containers, used tires) were collected in forest, savannah, barren and village land covers and flooded for eggs hatching. The emerging larvae were reared to adult, identified, and blood fed for F1 production. The F0 and F1 adults were identified and tested for ZIKV by Reverse Transcriptase-Real time Polymerase Chain Reaction.

**Results:**

A total of 1016 specimens, including 13 *Aedes* species, emerged in samples collected in the land covers and breeding sites investigated. *Ae. aegypti* was the dominant species representing 56.6% of this fauna with a high plasticity. *Ae. furcifer* and *Ae. luteocephalus* were found in forest tree holes, *Ae. taylori* in forest and village tree holes, *Ae. vittatus* in rock holes. ZIKV was detected from 4 out of the 82 mosquito pools tested. Positive pools included *Ae. bromeliae* (2 pools), *Ae. unilineatus* (1 pool), and *Ae. vittatus* (1 pool), indicating that the virus is maintained in these *Aedes* eggs during the dry season.

**Conclusion:**

Our investigation identified breeding sites types and land cover classes where several ZIKV vectors are maintained, and their maintenance rates during the dry season in southeastern Senegal. The maintenance of the virus in these vectors in nature could explain its early amplification at the start of the rainy season in this area.

## Background

Zika virus (ZIKV, genus *Flavivirus*, family *Flaviviridae*) was isolated for the first time in the Zika forest near Entebbe, Uganda, in 1947 from a febrile sentinel Rhesus monkey (*Macaca mulatta*). One year later the virus was isolated from *Aedes africanus* in the same area [1]. The first human case was reported in 1952 in Nigeria [2]. Subsequently, serological and virological studies showed the circulation of ZIKV in several other African [3] and Asian countries [4]. The virus was almost silent for 60 years until its reemergence in 2007 with the Yap Island and Gabon outbreaks [5]. It is during the last decade that the Asian genotype of ZIKV has experienced significant geographic expansion causing major epidemics in the Pacific Islands [6] and in America [7]. Indeed, phylogenic studies on sequenced ZIKV strains isolated from Brazil, Puerto Rico and Guatemala indicated that they were all within the Asian genotype and closely related to the French Polynesia strain [8, 9]. ZIKV is known to cause several debilitating neurological complications, including microcephaly in newborns and Guillain Barré Syndrome in adults, described in both Latin America and the Pacific Islands [10–13]. In the urban cycle, ZIKV is mainly transmitted by *Ae. aegypti* and *Ae. albopictus*, but cases of non-vector transmission like sexual transmission, blood transfusion, or other fluid transmissions were reported [7, 14, 15]. In West Africa, ZIKV has a sylvatic cycle (described in southeastern Senegal) involving mainly non-human primates and arboreal *Aedes (Ae. furcifer, Ae. luteocephalus, Ae. africanus, Ae. vittatus, Ae. dalzieli and Ae. taylori),* while *Ae. africanus* plays this role in Central and Eastern Africa [16, 17]. In this cycle, humans are rarely infected when entering in forest or by *Ae. furcifer* and *Ae. vittatus* in villages [17].

Evidences of ZIKV presence in human populations in Senegal were shown by the detection of the virus by serological and virological tests in samples collected from several localities (Dakar, Casamance, Ferlo, Diourbel, Sine Saloum, southeastern Senegal, Bandia, and Meckhé) between 1962 and 2015 [17–20]. In vectors, ZIKV was isolated in western Senegal from *Ae. luteocephalus* collected in 1968 in the Saboya forest [21] and from three mosquito species (*Aedes luteocephalus, Aedes furcifer-taylori* and *Anopheles gambiae*) in Bandia. As part of a program to study the biodiversity of arboviruses and their vectors in southeastern Senegal (1972–2015), more than 400 strains of ZIKV were isolated from around 20 species of mosquitoes belonging to the genera *Aedes*, *Anopheles*, *Culex* and *Mansonia* [17]. Recent studies have detected ZIKV from mosquitoes collected in different types of land covers including forests, savannahs, barrens, agricultures and villages across a large geographical area indicating wide spread of ZIKV in southeastern Senegal [17]. However, the main vectors belong to the genera *Aedes* (subgenera *Aedimorphus* (*Ae. dalzieli*), *Diceromyia* (*Ae. furcifer*, *Ae. taylori*) and *Stegomyia* (*Ae. luteocephalus*)) accounting for more than 90% of the isolates.

ZIKV is the most frequently amplified arbovirus in Senegal [12, 17, 21, 22]. Indeed, it was detected in mosquitoes during 21 of the 44 years of monitoring between 1972 and 2015 in the southeastern part of the country. However, ZIKV prevalence and vector density drop dramatically beyond detection by current methods each year during the dry season, generally between January and May. These data suggest that in southeastern Senegal, vectors and ZIKV are maintained locally in nature during adverse conditions corresponding to the dry season and the years when the virus is undetectable by current methods. Since the vectors belong mainly to the *Aedes* genus, their maintenance in nature is probably done through their desiccation-resistant eggs in larval breeding sites. The presence of eggs of 9 *Aedes* species from dry tree holes from one forest-gallery has already been demonstrated by Diallo and others in 1999–2000 [23]. However, the relative importance of tree holes for the dry season maintenance of these *Aedes* eggs compared to the others breeding sites [24] has not yet been investigated. The same is true of forests compared to other land covers. The detection of the virus in a pool of male *Ae. furcifer* in 2011 [17] suggests that vertical transmission, from infected females to its progeny via eggs, may be an important mechanism for local virus maintenance. In addition, there is currently no data on the mechanisms and vertical transmission rates in nature. There is also no data on species and breeding sites involved and their levels of participation in this mechanism. Our first objective was to identify the vector species in which Zika virus is maintained during dry season, and their specific infection rates, using genetic testing methods. Our second objective was to identify if vector species are more likely to use certain combinations of breeding site types and land cover classes than others to lay their eggs, using mainly a qualitative statistical approach on vector species occurrence. We expected that Zika virus would be maintained through certain mosquito species, and these mosquito species preferentially use certain combinations of breeding site types and land cover classes to lay their eggs.

## Methods

### Sampling sites

**Fig. 1:**
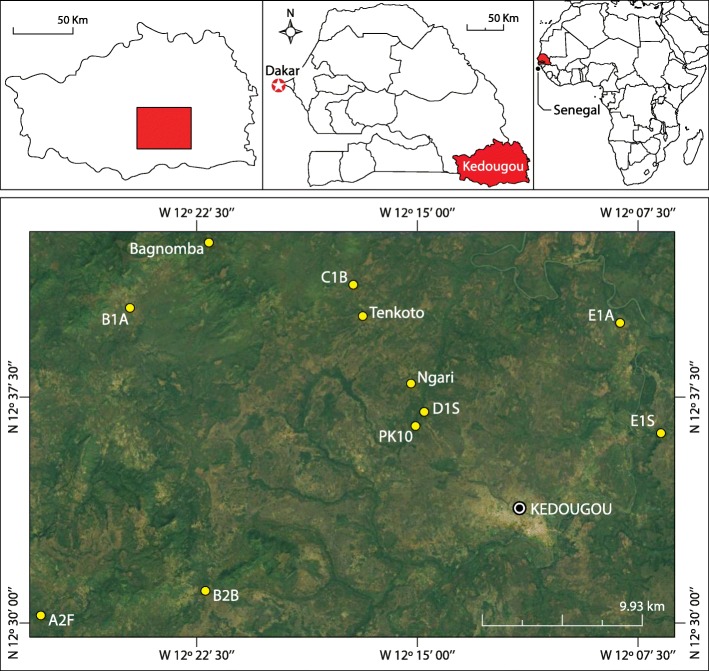
Map showing different *Aedes* egg sampling sites in the Kédougou in December 2015, October 2016 and March 2017. Insets are obtained from ArcGIS Desktop V 10.5 (ESRI 2019, Redlands, CA: Environmental Systems Research Institute; https://desktop.arcgis.com/en/); sampling sites are placed on base map using their respective XY coordinates and ArcGIS Desktop. Base map was obtained from Google earth: Google earth V 7.3.2.5776. (December 14, 2015). Kedougou, Senegal, Image© 2019 Maxar Technologies, Image© 2019 CNES / Airbus, US Dept of State Geographer, http://www.earth.google.com [April 17, 2019]

The study was undertaken in the Kédougou area, located 702 km from Dakar in the extreme south-east of Senegal (Fig. 1). It belongs to the Sudano-Guinean phytogeographical zone. It is an ecotone between a tropical dry forest and a savannah area favorable to the circulation of several arboviruses such as ZIKV and dengue virus. It is the rainiest region of Senegal (isohyets between 900 and 1600 mm) [25] with a dense hydrographic network, and a highly diversified fauna and flora.

### Samples collection in the field

Samples were collected in December 2015, October 2016 and March 2017 corresponding to the beginning and middle of the dry season in Kédougou during these years. Collection of soil, sand and detritus from dry breeding sites of arbovirus vectors was done using spoons and washing of the breading sites and water recovery. The known *Aedes* vectors breeding sites in the area [24] including fruit husks, tree holes, rock holes, discarded containers, and used tires were sampled in 3 villages (Ngari, Bagnomba, and Tenkoto), 2 forests galleries (PK10 and A2F), 2 savannahs (D1S and E1S), 2 barrens (C1B and B2B), and 2 agricultures (B1A and E1A) (Fig. 1). These land covers were identified and well described in several previous papers [26, 27]. Samples were put in individual plastic bags or boxes and transported to the Laboratory of the medical zoology pole of Institut Pasteur de Dakar. No permission was needed for samples collection.

### Sample processing in the laboratory

Soil samples were flooded in tap water for eggs hatching and placed in a room with optimal temperature conditions for larval growth (29 to 30 °C). The boxes were checked daily to monitor hatching and larval growth. Pupae were recovered and placed in emergence pots covered by a mosquito net. This process was repeated 3 times for each sample in order to have the maximum egg hatching. After emergence, adult mosquitoes were identified using appropriate dichotomous identification keys [28, 29].

This F0 generation was maintained under standard insectarium conditions [30] (temperature of 27 ± 1 °C, relative humidity of 80% and a photoperiod of 12: 12 h) and was fed with 10% sucrose and then authorized to take a blood meal for egg production 1 week after emergence. These eggs were then flooded and the hatching larvae reared to F1 adults at the standard insectarium conditions described above. F0 and F1 mosquito adults were frozen, identified and pooled in 1 to 50 individuals by species, breeding site and land cover of origin, and kept at − 80 °C for ZIKV detection attempts by Reverse Transcriptase-Real time Polymerase Chain Reaction (RT-PCR).

### Virus detection

Mosquitoes were triturated using cold pestles in 500 μl of L-15 medium (GibcoBRL, Grand Island, NY, USA). After trituration, pools were centrifuged at 7500 rpm for 10 min at 4 °C. For each sample, 100 μl of supernatant were used for RNA extraction with the QiaAmp Viral RNA Extraction Kit (Qiagen, Heiden, Germany) according to the manufacturer’s protocol with slight modification [31]. RNA was amplified using a real-time RT-PCR assay and an ABI Prism 7500 SDS Real-Time apparatus (Applied Biosystems, Foster City, CA) using the QuantiTect kit (Qiagen, Hilden, Germany). The 25 μl reaction volume contained 5 μl of extracted RNA, 10 μl of buffer (2x QuantiTect Probe), 6.8 μl of RNase free water, 1.25 μl of each primer, 0.5 μl of probe, and 0.2 μl of enzymes. Primers ZIKV 835 (TTGGTCATGATACTGCTGATTGC) and ZIKV 911c (CCTTCCACAAAGTCCCTATTGC) and probe ZIKV 860-FAM (CGGCATACAGCATCAGGTGCATAGGAG), described by Lanciotti et al. [32] were used.

### Data analysis

For each species, relative abundances and maintenance rates in the different breeding site types and land covers were calculated as well as the minimum infection rate for species found positive for ZIKV. Relative abundance for a given species represents the percentage of the number of emerging individuals of this species to the total number of individuals hatched from the same breeding site type of the same land cover. The maintenance rate for each species corresponds to the percentage of the number of positive breading site type for this species to the total number of breeding sites of the same land cover flooded. The effect of breeding site (tree holes, rock holes, discarded containers, and used tires) and land cover types (forests, savannahs, barrens, villages, and agricultures) on each of the species vector abundance was analyzed using a generalized linear mixed-effect model (GLMM), using sampling month as random factors, with Poisson error distribution. Tukey’s post hoc test was used to identify significant individual comparisons. The minimum field infection rate (MFIR) for each species is the percentage of the number of positive mosquito pools of this species to the total number of mosquitoes of this species tested. The chi-2 test was used to compare relative abundances and maintenance rates of each of major vectors in different breeding site types from the different land covers and infection rates of different species. Differences were considered statistically significant at *p* < 0.05. The statistical tests were performed using R software [33].

## Results

### Mosquito species composition and relative abundance in different breeding sites

A total of 1016 mosquitoes belonging to 13 species of the genera *Aedes* were collected at our study sites in December 2015, October 2016, and March 2017 (Table 1). Adult mosquitoes emerged from eggs collected in forest (734 specimens), savannah (77 specimens), agriculture (7 specimens), village (163 specimens) and barren (35 specimens) land covers. A greater diversity of the mosquito fauna was observed in the forests (Table 1) with a total of 12 species collected in this land cover*.* The forests were followed by savannahs, villages, and barrens with 5 species collected in each land cover. Agricultures had the lowest diversity with only 2 species collected. Tree holes were the breeding sites with the more diversified mosquito fauna (Table 1) with 9 species collected. They were followed by rock holes with 8 species and tires with 3 species. The least diversified breeding sites were discarded containers where only 2 species were collected.

**Table 1:** Specific composition and relative abundance of *Aedes* species collected in different land covers classes and breeding sites in December 2015, October 2016 and March 2017 in Kédougou

Breeding sites	Species	Land Covers										TOTAL	
		Forest		Savanah		Agriculture		Village		Barren			
		No.	%	No.	%	No.	%	No.	%	No.	%	No.	%
	*Ae. aegypti*	91	52.9	10	13	0	0	111	74.5	0	0	212	52.3
	*Ae. bromeliae*	17	9.9	12	15.6	6	85.7	34	22.8	0	0	69	17
	*Ae. fowleri*	0	0	1	1.3	0	0	0	0	0	0	1	0.2
	*Ae. furcifer*	3	1.7	0	0	0	0	0	0	0	0	3	0.7
Tree holes	*Ae. longipalpis*	11	6.4	0	0	0	0	0	0	0	0	11	2.7
	*Ae. luteocephalus*	13	7.6	0	0	0	0	0	0	0	0	13	3.2
	*Ae. stokesi*	1	0.6	2	2.6	0	0	1	0.7	0	0	4	1
	*Ae. taylori*	7	4.1	0	0	0	0	1	0.7	0	0	8	2
	*Ae. unilineatus*	29	16.9	52	67.5	1	14.3	2	1.3	0	0	84	20.7
Total mosquitoes		172		77		7		149		0		405	
	*Ae. aegypti*	214	43.3	0	0	0	0	0	0	23	65.7	237	44.8
	*Ae. bromeliae*	1	0.2	0	0	0	0	0	0	9	25.7	10	1.9
	*Ae. dalzieli*	0	0	0	0	0	0	0	0	1	2.9	1	0.2
	*Ae. fowleri*	5	1	0	0	0	0	0	0	0	0	5	0.9
Rock holes	*Ae. hirsutus*	39	7.9	0	0	0	0	0	0	0	0	39	7.4
	*Ae. minutus*	38	7.7	0	0	0	0	0	0	1	2.9	39	7.4
	*Ae. taylori*	0	0	0	0	0	0	0	0	1	2.9	1	0.2
	*Ae. vittatus*	197	39.9	0	0	0	0	0	0	0	0	197	37.2
Total mosquitoes		494								35		529	
Discarded containers	*Ae. aegypti*	0	0	0	0	0	0	6	54.5	0	0	6	54.5
	*Ae. bromeliae*	0	0	0	0	0	0	5	45.5	0	0	5	45.5
Total mosquitoes		0		0		0		11		0		11	
	*Ae. aegypti*	66	97	0	0	0	0	3	100	0	0	69	97.2
Used tires	*Ae. bromeliae*	1	1.5	0	0	0	0	0	0	0	0	1	1.4
	*Ae. luteocephalus*	1	1.5	0	0	0	0	0	0	0	0	1	1.4
Total mosquitoes		68		0		0		3		0		71	
	*Ae. aegypti*	371	50.5	10	13	0	0	120	73.6	23	65.7	524	51.6
	*Ae. bromeliae*	19	2.6	12	15.6	6	85.7	39	23.9	9	25.7	85	8.4
	*Ae. dalzieli*	0	0	0	0	0	0	0	0	1	2.9	1	0.1
Total breading sites	*Ae. fowleri*	5	0.7	1	1.3	0	0	0	0	0	0	6	0.6
	*Ae. hirsutus*	39	5.3	0	0	0	0	0	0	0	0	39	3.8
	*Ae. furcifer*	3	0.4	0	0	0	0	0	0	0	0	3	0.3
	*Ae. longipalpis*	11	1.5	0	0	0	0	0	0	0	0	11	1.1
	*Ae. luteocephalus*	14	1.9	0	0	0	0	0	0	0	0	14	1.4
	*Ae. minutus*	38	5.2	0	0	0	0	0	0	1	2.9	39	3.8
	*Ae. stokesi*	1	0.1	2	2.6	0	0	1	0.6	0	0	4	0.4
	*Ae. taylori*	7	1	0	0	0	0	1	0.6	1	2.9	9	0.9
	*Ae. unilineatus*	29	4	52	67.5	1	14.3	2	1.2	0	0	84	8.3
	*Ae. vittatus*	197	26.8	0	0	0	0	0	0	0	0	197	19.4
	Total mosquitoes	734		77		7		163		35		1016	

*Ae. aegypti* was the dominant species, accounting for 51.6% of this fauna (Table 1). It was followed in descending order, among the potential vectors of ZIKV, by *Ae. vittatus* (19.4%), *Ae. bromeliae* (8.4%), *Ae. unilineatus* (8.3%), *Ae. hirsutus* (3.8%), *Ae. luteocephalus* (1.4%), *Ae. taylori* (0.9%), *Ae. fowleri* (0.6%), *Ae. furcifer* (0.3%) and *Ae. dalzieli* (0.1%). The dominant species varied according to the breeding site and land cover (Table 1). Thus, *Ae. aegypti* was the dominant species in tree holes from forests (52.9%) and villages (74.5%), rock holes from forests (43.3%) and barrens (65.7%), tires from forests (97.1%) and discarded containers from villages (54.5%). This species was the only one present in tires from villages. The relative abundance of *Ae. aegypti* in different tree holes from all land cover were significantly different (*p* < 0.001).

### Maintenance patterns of mosquito species in different breeding sites

A total of 1339 samples, including 687 tree holes, 462 rock holes, 71 discarded containers, 29 used tires and 90 fruit husks of *Saba senegalensis* were collected and flooded for eggs hatching. No positive breeding site was noted for fruit husks. The maintenance rates of vectors in the different land cover classes and breeding sites are presented in Table 2. *Ae. aegypti* was the species that persisted in the largest number of breeding sites in the different land covers investigated. Adults of this species emerged in tires from forests (66.7%, 2 out of 3 flooded sites), and villages (7.7%, 2/26), rock holes from forests (7.4%, 20/270), and barrens (1%, 2/192), tree holes from forests (6%, 15/251), villages (4.7%, 7/149), and savannahs (1.8%, 4/227), and discarded containers from villages (2.8%, 2/71). These rates showed statistically significant differences (*p* < 0.001). However, maintenance rates of this species in rock and tree holes from forests, discarded containers, tires and tree holes from villages were comparable (*p* = 0.05). The other potential vectors were less plastic. Even if *Ae. unilineatus* was only found in tree holes, this vector was found in this breading site from forests (0.4%, 1/227), savannahs (0.4%, 1/227), agricultures (0.4%, 1/227) and villages (0.4%, 1/227). *Ae. fowleri* and *Ae. taylori* were found in tree and rock holes of different land covers. Indeed, *Ae. fowleri* viable eggs were maintained in tree holes from savannahs (0.4%, 1/227) and forests (0.7%, 2/270) while *Ae. taylori* eggs were maintained in tree holes from forests (2%, 5/251) and villages (0.7%, 1/149) and in rock holes from forests (0.4%, 1/270) and barrens (0.5%, 1/192). *Ae. luteocephalus* was maintained only in tree holes (2%, 5/251) and tires (33.3%, 1/3) from forests. For each of these species, the maintenance rates in the different breeding sites were comparable (*p* > 0.07). The only species that was maintained in only one type of breeding site and land cover were *Ae. furcifer* in tree holes from forests (0.8%, 2/251) and *Ae. vittatus* in rock holes from forests (11.1%, 30/270).

**Table 2:** Maintenance rates of potential Zika virus vectors in the different breeding sites and land cover classes collected in December 2015, October 2016 and March 2017 in the Kédougou region

Breeding sites	Species	Land covers										TOTAL	
		Forest		Savannah		Agriculture		Village		Barren			
		BS+	%	BS+	%	BS+	%	BS+	%	BS+	%	BS+	%
Tree holes	*Ae. aegypti*	15	6	4	1.8	0	0	7	4.7	0	0	26	3.8
	*Ae. fowleri*	0	0	1	0.4	0	0	0	0	0	0	1	0.1
	*Ae. furcifer*	2	0.8	0	0	0	0	0	0	0	0	2	0.3
	*Ae. luteocephalus*	5	2	0	0	0	0	0	0	0	0	5	0.7
	*Ae. taylori*	5	2	0	0	0	0	1	0.7	0	0	6	0.9
	*Ae. unilineatus*	13	5.2	10	4.4	1	1.7	2	1.3	0	0	26	3.8
Total flooded		251		227		60		149		0		687	
Rock holes	*Ae. aegypti*	20	7.4	0	0	0	0	0	0	2	1	22	4.8
	*Ae. dalzieli*	0	0	0	0	0	0	0	0	1	0.5	1	0.2
	*Ae. fowleri*	2	0.7	0	0	0	0	0	0	0	0	2	0.4
	*Ae. hirsutus*	1	0.4	0	0	0	0	0	0	0	0	1	0.2
	*Ae. taylori*	1	0.4	0	0	0	0	0	0	1	0.5	2	0.4
	*Ae. vittatus*	30	11.1	0	0	0	0	0	0	0	0	30	6.5
Total flooded		270		0		0		0		192		462	
Discarded containers	*Ae. aegypti*	0	0	0	0	0	0	2	2.8	0	0	2	2.8
Total flooded		0		0		0		71		0		**71**	
Used tires	*Ae. aegypti*	2	66.7	0	0	0	0	2	7.7	0	0	4	13.7
	*Ae. luteocephalus*	1	33.3	0	0	0	0	0	0	0	0	1	3.4
Total flooded		3		0		0		26		0		29	

*BS+* Number of positive Breeding sites

### Generalized linear mixed model for mosquito abundance

Results of the GLMM indicated that breeding site, and landcover types did not affect the abundance of *Ae. bromeliae*, and *Ae. taylori* (Table 3; *p* > 0.05). The model also revealed that there was no significant difference in abundance of *Ae. aegypti* in land cover classes (*p* < 0.03). The abundance of *Ae. aegypti* in tires was significantly higher than in DC, RH and TH (*P* < 0.0001). The abundance of *Ae. unilineatus* were similar in agriculture, barren, forest and village (*p* > 0.16), and significantly lower than in Savannah land cover (*p* < 0.03).

**Table 3:** Abundance of species potential vectors of Zika virus potential vectors in the different breeding sites and land cover classes collected in December 2015, October 2016 and March 2017 in the Kédougou region

		Species									
		*Ae. aegypti*	*Ae. vittatus*	*Ae. bromeliae*	*Ae. unilineatus*	*Ae. hirsutus*	*Ae. luteocephalus*	*Ae. taylori*	*Ae. fowleri*	*Ae. furcifer*	*Ae. dalzieli*
Breeding sites	Used tires	0.28 (1.4)^a^	0 (0)	0.03 (0.14)^a^	0 (0)	0 (0)	0.03 (0.14)	0 (0)	0 (0)	0 (0)	0 (0)
	Discarded containers	0.03 (0.26)^b^	0 (0)	0 (0)^a^	0 (0)	0 (0)	0 (0)	0 (0)	0 (0)	0 (0)	0 (0)
	Tree holes	0.07 (0.43)^b^	0 (0)	0.04 (0.26)^a^	0.04 (0.28)	0 (0)	0.008 (0.11)	0.002 (0.04)	0.001 (0.03)	0.003 (0.51)	0 (0)
	Rock holes	0.09 (0.57)^b^	0.12 (0.58)	0.009 (0.12)^a^	0 (0)	0.012 (0.2)	0 (0)	0.002 (0.03)	0.005 (0.08)	0 (0)	0.002 (0.3)
Land cover classes	Agriculture	0 (0)^a^	0 (0)	0.03 (0.28)^a^	0.01 (0.09)^a^	0 (0)	0 (0)	0 (0) ^a^	0 (0)	0 (0)	0 (0)
	Barren	0.03 (0.29)^a^	0 (0)	0.02 (0.18)^a^	0 (0)^a^	0 (0)	0 (0)	0.004 (0.05) ^a^	0 (0)	0 (0)	0 (0)
	Forest	0.14 (0.69)^a^	0.11 (0.54)	0.02 (0.15)^a^	0.03 (0.19)^a^	0.01 (0.19)	0.1 (0.13)	0.001 (0.03) ^a^	0.005 (0.08)	0.004 (0.59)	0 (0)
	Savannah	0.02 (0.18)^a^	0 (0)	0.03 (0.19)^a^	0.07 (0.41)^b^	0 (0)	0 (0)	0 (0) ^a^	0.003 (0.05)	0 (0)	0 (0)
	Village	0.07 (0.49)^a^	0 (0)	0.03 (0.3)^a^	0.006 (0.06)^a^	0 (0)	0 (0)	0.003 (0.05) ^a^	0 (0)	0 (0)	0 (0)

Mean number (and standard deviation) of mosquito eggs collected in different breeding sites and land cover classes are presented. For each species, breeding sites and land cover with different superscript letters are significantly different. Tests were done only when more than 2 sites or land cover classes have eggs

### Minimum field infection rate of ZIKV in potential vectors

A total of 1768 mosquitoes (748 males and 1020 females, 82 pools) were tested for ZIKV infection by RT-PCR. Four (4) of the 28 pools of F0 generation collected in March 2017 were positive for ZIKV. Positive mosquitoes consisted of 2 pools of *Ae. bromeliae* females (cycle thresholds: 39.53 and 34.79), 1 pool of *Ae. unilineatus* females (ct: 33.02) and 1 pool of *Ae. vittatus* males (ct:34.83) (Table 4). Mosquitoes were collected in tree holes from villages (20 females) and rock holes from forests (4 females) for *Ae. bromeliae*, in tree holes from savannahs for *Ae. unilineatus* (20 females) and rock holes from forests for *Ae. vittatus* (8 males). MFIR of the infected species varied between 5% for *Ae. bromeliae* and *Ae. unilineatus* in the breeding site and landcovers where they were positives to 25% for *Ae. bromeliae* females collected from rock holes in forest. The MFIR of the infected species in the different land covers classes were statistically similar (*p* = 0.7).

**Table 4:** Minimum infection rate of species infected with Zika virus collected in the Kédougou in March 2017 in different land covers and breeding sites

Breeding sites	Species	Sex		Land covers		
				Forest	Savannah	Village
Tree holes	*Aedes bromeliae*	female	P(T)	–	–	1 (20)
			MFIR			5.0
	*Ae. unilineatus*	female	P(T)	–	1 (20)	–
			MFIR		5.0	
Rock holes	*Ae. bromeliae*	female	P(T)	1 (4)	–	–
			MFIR	25.0		
	*Ae. vittaus*	male	P(T)	1 (8)	–	–
			MFIR	12.5		

*P* (*T*) Number of positive pools (Total mosquitoes tested)

*MFIR* Minimum field infection rate (% of infected mosquitoes)

## Discussion

Our study showed that 13 *Aedes* species eggs were maintained in the different land covers and breeding sites sampled in southeastern Senegal. This diversity is greater than that observed in a previous study conducted in the same area with only 9 *Aedes* species emerged from eggs samples collected in tree holes [23]. The higher number of breeding sites and land cover classes investigated in our study would explain this difference. Indeed, Diallo and others [23] only surveyed tree holes from a single forest gallery while our study covered 4 types of breeding sites in 5 different land cover classes. However, 12 *Aedes* species previously found as larvae [24] or adults [27] in this area were not found in this study. This could be explained by the inaccessibility of tree holes located at height higher than 2 m, which may be preferential habitat for these arboreal species. Indeed, variations of breeding behavior of some mosquito species depending on tree height were observed by some authors [34, 35]. The entrances of these tree holes may also be hidden by their small sizes or their location behind tree barks. Despite the fact that we were interested to the eggs (that are not mobile), sampling in different months and years could be considered as a limitation in our study.

Our study also showed that fruit husks were not involved in vectors maintenance during the dry season. This result was not expected because between 89 and 100% of these fruit husks were positives for larvae or pupae of 8 *Aedes* species in a previous study in the same area [24]. The particular physical, chemical and biological conditions of water contained in these fruit husks could alter the potential of resistance of *Aedes* eggs to desiccation resulting in the destruction of all eggs that did not hatch during rainy season. Further investigations on conditions that prevent fruit husks from being used as *Aedes* eggs maintenance sites could ultimately lead to the identification of substances or organisms that can be used for eggs destruction, and thus, in controlling major arboviruses of medical importance such as Zika, dengue, yellow fever and chikungunya. Maintenance rates were quite low compared to larval presence rates during the rainy season for all vectors listed, indicating an impact of the climatic conditions on the survival of *Aedes* eggs [36]. Indeed, even if *Aedes* eggs are resistant to desiccation, their viability decreases with time. The low relative humidity of the air also reduces survival time of *Aede*s eggs [37, 38]. In addition, the proportion of resistant eggs during a given period is greater in shaded sites than those exposed to the sun [39].

The dominant species *Ae. aegypti* was present in almost all breeding sites and land covers sampled. The plasticity of this species observed in our study is consistent with larval survey data in the area and elsewhere [24, 40] but also with its oviposition behavior in Ivory Coast [41]. This greater plasticity of *Ae. aegypti* suggests that its eggs are more resistant to desiccation and lower humidity than other species [37, 42].

Finding viable eggs of *Ae. taylori* maintained in villages was not expected. Indeed, this species was almost never found biting humans within villages area [17, 26]. This species could feed on other animals within villages or breed in some villages close to wild environment before going back to forests and savannahs where it bites. The localized maintenance of *Ae. furcifer* in tree holes from forests is consistent with its oviposition behavior but discordant with the fact that it could feed in all the land covers found in the area including villages indoors [17].

Viable eggs of *Ae. vittatus* were maintained in rock holes of forests in concordance with its breeding preference in Nigeria [43]. Nevertheless, larval survey data in the area and elsewhere have shown that the species can colonize a wide range of breeding sites and land cover classes [24, 44]. The maintenance of *Ae. vittatus* exclusively in rock holes from forests could be explained by total egg mortality in other land covers that are exposed to very high temperatures associated with low relative humidity. Rock holes from forests in which *Ae. vittatus* was maintained were relatively shady and filled with dead leaves creating a microclimate that is less warm, wetter and thus more favorable to eggs survival of this species.

ZIKV detection by RT-PCR in 4 mosquito pools from 3 different species indicating for the first time that the virus can be maintained locally during dry season in these potential vectors through vertical transmission. This possibility had already been suggested by detection of the virus from a male *Ae. furcifer* in the area [17]. A recent study detected ZIKV in 5 adults *Ae. albopictus* emerged from eggs collected in 2015 in Bahia, Brazil [45]. In addition, vertical transmission of ZIKV has also been proven for *Ae. aegypti* and *Ae. albopictus* in laboratory studies [46, 47]. The maintenance of ZIKV in 3 species in southeastern Senegal explains its frequent amplification from the beginning of the rainy season. Although these vertical transmission rates of arboviruses are generally low, they are of great epidemiological importance in nature. Indeed, they allow prolonged conservation of viruses during unfavorable periods to transmission (dry season) where vectors live in state of eggs and sensitive hosts are rare. The implication of *Ae. vittatus* in ZIKV maintenance strengthens its status of potential epidemic vector. Indeed, *Ae. vittatus* has already been found associated with ZIKV in nature and competent for the virus in laboratory [17, 48]. Our study revealed for the first time the association of *Ae. bromeliae* with ZIKV in the area. Particular attention should be paid to this vector belonging to the same group as *Ae. simpsoni* which is one of the main vectors of yellow fever in East Africa [49]. Nevertheless, further studies are needed on the vector competence of this species for ZIKV. *Aedes unilineatus* has already been found associated to ZIKV in nature and able to disseminate but not to transmit the virus [17, 48]. Its implication in maintaining ZIKV suggests that it may also play a more important epidemiological role in ZIKV epidemiology in Africa.

## Conclusion

This study allows us to identify breeding sites, land cover classes and maintenance rates of several important ZIKV vectors in southeastern Senegal. It has also highlighted ZIKV maintenance in the area as well as species involved and breeding sites and land covers in which virus is maintained. These results provide a better understanding of the epidemiology of ZIKV disease in southeastern Senegal.

## Data Availability

All data generated or analyzed during this study are included in this published article.

## Abbreviations

ZIKV: Zika virus
MFIR: Minimum field Infection Rate BS+ = Number of positive Breeding sites
MFIR: Minimum field infection rate
GLMM: Generalized linear mixed-effect model
P (T): Number of positive pools (Total mosquitoes tested)
*Ae.*: *Aedes*
*Cx.*: *Culex*
*An.*: *Anopheles*
*Ma.*: *Mansonia*

## References

[1] Dick GWA, Kitchen SF, Haddow AJ (1952). Zika Virus (I). Isolations and serological specificity. Trans R Soc Trop Med Hyg..

[2] MacNamara FN (1954). Zika virus : A report on three cases of human infection during an epidemic of jaundice in Nigeria. Trans R Soc Trop Med Hyg..

[3] Bres P (1970). Donnees recentes apportees par les enquetes serologiques sur la prevalence des arbovirus en Afrique, avec reference spéciale à la fievre jaune. Bull World Health Organ..

[4] Hammon WM, Schrack WD, Sather GE (1958). Serological Survey for Arthropod-Borne Virus Infections in the Philippines1. Am J Trop Med Hyg..

[5] Grard G, Caron M, Mombo IM, Nkoghe D, Mboui Ondo S, Jiolle D (2014). Zika Virus in Gabon (Central Africa) – 2007: A New Threat from *Aedes albopictus*?. PLoS Negl Trop Dis..

[6] Roth A, Mercier A, Lepers C, Hoy D, Duituturaga S, Benyon E (2014). Concurrent outbreaks of dengue, chikungunya and Zika virus infections – an unprecedented epidemic wave of mosquito-borne viruses in the Pacific 2012–2014. Euro Surveill..

[7] Safadi MA, Nascimento-Carvalho CM (2017). Update on Zika: What You Need to Know. Pediatr Infect J..

[8] Zanluca C, Melo VC, Mosimann AL, Santos GI, Santos CN, Luz K (2015). First report of autochthonous transmission of Zika virus in Brazil. Mem Inst Oswaldo Cruz..

[9] Lanciotti RS, Lambert AJ, Holodniy M, Saavedra S, Signor LC (2016). Phylogeny of Zika Virus in Western Hemisphere, 2015. Emerg Infect Dis..

[10] Cauchemez S, Besnard M, Bompard P, Dub T, Guillemette-Artur P, Eyrolle-Guignot D (2016). Association between Zika virus and microcephaly in French Polynesia, 2013-15: a retrospective study. Lancet..

[11] Capasso A, Ompad DC, Vieira DL, Wilder-Smith A, Tozan Y (2019). Incidence of Guillain-Barré Syndrome (GBS) in Latin America and the Caribbean before and during the 2015-2016 Zika virus epidemic: A systematic review and meta-analysis. PLoS Negl Trop Dis..

[12] Musso D, Gubler DJ (2016). Zika Virus. Clin Microbiol Rev..

[13] Mlakar J, Korva M, Tul N, Popovic M, Poljsak-Prijatelj M, Mraz J (2016). Zika Virus Associated with Microcephaly. N Engl J Med..

[14] Grischott F, Puhan M, Hatz C, Schlagenhauf P (2016). Non-vector-borne transmission of Zika virus: A systematic review. Travel Med Infect Dis..

[15] Baud D, Musso D, Vouga M, Alves MP, Vulliemoz N (2017). Zika virus: A new threat to human reproduction. Am J Reprod Immunol..

[16] Boyer S, Calvez E, Chouin-Carneiro T, Diallo D, Failloux AB (2018). An overview of mosquito vectors of Zika virus. Microbes Infect..

[17] Diallo D, Sall AA, Diagne CT, Faye O, Faye O, Ba Y (2014). Zika Virus Emergence in Mosquitoes in Southeastern Senegal, 2011. Attoui H, editor. PLoS ONE.

[18] Bres P, Lacan A, Michel R, Peretti P, Vidal C (1963). Arboviruses in Senegal. A Virological Survey. Bull Soc Pathol Exot..

[19] Bres P, Lacan A, Diop I, Michel R, Peretti P, Vidal C (1963). Arborviruses in Senegal. Serological Survey. Bull Soc Pathol Exot Fil..

[20] Renaudet J, Jan C, Ridet J, Adam C, Robin Y (1978). A serological survey of arboviruses in the human population of Senegal. Bull Soc Pathol Exot Fil..

[21] Cornet M, Robin Y, Chateau R, Hème G, Adam C, Valade M (1979). Isolements d’arbovirus au Sénégal oriental apartir de moustiques (1972–1977) et notes sur l’épidémiologie des virus transmis par les *Aedes*, en particulier du virus amaril. Cah ORSTOM, Sér Ent Méd Parasitol..

[22] Adam F, Diguette JP. Virus d’Afrique [base de données]. Dakar: Institut Pasteur de Dakar. Centre collaborateur OMS de référence et de recherche pour les arbovirus et les virus de fièvres hémorrhagiques (CRORA). 2005.

[23] Diallo M, Ba Y, Sall AA, Diop OM, Ndione JA, Mondo M (2003). Amplification of the Sylvatic Cycle of Dengue Virus Type 2, Senegal, 1999–2000: Entomologic Findings and Epidemiologic Considerations. Emerg Infect Dis..

[24] Diallo D, Diagne CT, Hanley KA, Sall AA, Buenemann M, Ba Y (2012). Larval ecology of mosquitoes in sylvatic arbovirus foci in southeastern Senegal. Parasit Vectors..

[25] ANDS/ RGPHAE-Sénégal, Agence Nationale de la Statistique et de la Démographie. http://www.ansd.sn/index.php, Accessed 6 Mar 2017.

[26] Diallo D, Sall AA, Buenemann M, Chen R, Faye O, Diagne CT (2012). Landscape ecology of sylvatic chikungunya virus and mosquito vectors in southeastern Senegal. PLoS Negl Trop Dis..

[27] Richman R, Diallo D, Diallo M, Sall AA, Faye O, Diagne CT (2018). Ecological niche modeling of Aedes mosquito vectors of chikungunya virus in southeastern Senegal. Parasit Vectors..

[28] Edwards FW. Mosquitoes of the Ethiopian Region. III.-Culicine adults and pupae. Mosquitoes Ethiop Reg III-Culicine Adults Pupae. 1941.

[29] Ferrara L, Germain M, Hervy J-P (1984). *Aedes* (*Diceromyia*) *furcifer* (Edwards, 1913) et *Aedes* (*Diceromyia*) *taylori* Edwards, 1936: le point sur la différenciation des adultes. Cah ORSTOM, Sér Ent Méd Parasitol..

[30] Gerberg EJ, Barnard DR, Ward RA. Manual for mosquito rearing and experimental techniques. Bulletin No. 5. Lake Charles: American Mosquito Control Association; 1994.

[31] Faye O, Faye O, Diallo D, Diallo M, Weidmann M, Sall A (2013). Quantitative real-time PCR detection of Zika virus and evaluation with field-caught Mosquitoes. Virol J..

[32] Lanciotti RS, Kosoy OL, Laven JJ, Velez JO, Lambert AJ, Johnson AJ (2008). Genetic and Serologic Properties of Zika Virus Associated with an Epidemic, Yap State, Micronesia, 2007. Emerg Infect Dis..

[33] R core Team (2016). R: A language and environment for statistical computing.

[34] Service MW (1965). The Ecology of the Tree-Hole Breeding Mosquitoes in the Northern Guinea Savanna of Nigeria. J Appl Ecol..

[35] Lounibos LP (1981). Habitat segregation among African treehole mosquitoes. Ecol Entomol..

[36] Costa EA, Santos EM, Correia JC, Albuquerque CM (2010). Impact of small variations in temperature and humidity on the reproductive activity and survival of *Aedes aegypti* (Diptera, Culicidae). Rev Bras Entomol..

[37] Sota T, Mogi M (1992). Survival time and resistance to desiccation of diapause and non-diapause eggs of temperate *Aedes* (*Stegomyia*) mosquitoes. Entomol Exp Appl..

[38] Roberts D (2004). Prolonged survival of eggs of the rock-pool mosquito, *Aedes vittatus*, in the extreme heat of the Arabian Peninsula. J Arid Environ..

[39] Cordellier R, Germain M, Mouchet J (1974). Les vecteurs de fièvre jaune en Afrique. Cah ORSTOM, Sér Ent Méd Parasitol..

[40] Simard FDR, Nchoutpouen EE, Toto JC, Fontenille D (2005). Geographic Distribution and Breeding Site Preference of *Aedes albopictus* and *Aedes aegypti* (Diptera: Culicidae) in Cameroon, Central Africa. J Med Entomol..

[41] Zahouli JBZ, Utzinger J, Adja MA, Müller P, Malone D, Tano Y (2016). Oviposition ecology and species composition of *Aedes* spp. and *Aedes aegypti* dynamics in variously urbanized settings in arbovirus foci in southeastern Côte d’Ivoire. Parasit Vectors.

[42] Juliano SA, O’Meara GF, Morrill JR, Cutwa MM (2002). Desiccation and thermal tolerance of eggs and the coexistence of competing mosquitoes. Oecologia..

[43] Adebote DA, Oniye SJ, Muhammed YA (2008). Studies on mosquitoes breeding in rock pools on inselbergs around Zaria, northern Nigeria. J Vector Borne Dis..

[44] Tewari SC, Thenmozhi V, Katholi CR, Manavalan R, Munirathinam A, Gajanana A (2004). Dengue vector prevalence and virus infection in a rural area in south India. Trop Med Int Health..

[45] Smartt CT, Stenn TMS, Chen T-Y, Teixeira MG, Queiroz EP, Souza Dos Santos L (2017). Evidence of Zika Virus RNA Fragments in *Aedes albopictus* (Diptera: Culicidae) Field-Collected Eggs From Camaçari, Bahia, Brazil. J Med Entomol..

[46] Thangamani S, Huang J, Hart CE, Guzman H, Tesh RB (2016). Vertical Transmission of Zika Virus in *Aedes aegypti* Mosquitoes. Am J Trop Med Hyg..

[47] Ciota AT, Bialosuknia SM, Ehrbar DJ, Kramer LD (2017). Vertical Transmission of Zika Virus by *Aedes aegypti* and *Ae. albopictus* Mosquitoes. Emerg Infect Dis..

[48] Diagne CT, Diallo D, Faye O, Ba Y, Faye O, Gaye A (2015). Potential of selected Senegalese *Aedes* spp. mosquitoes (Diptera: Culicidae) to transmit Zika virus. BMC Infect Dis..

[49] Huang Y-M (1986). *Aedes* (*Stegomyia*) *bromeliae* (Diptera: Culicidae), the Yellow Fever Virus Vector in East Africa1. J Med Entomol..

